# Two novel *Bartonella* (sub)species isolated from edible dormice (*Glis glis*): hints of cultivation stress-induced genomic changes

**DOI:** 10.3389/fmicb.2023.1289671

**Published:** 2023-11-15

**Authors:** Oldřich Bartoš, Běla Klimešová, Karolina Volfová, Martin Chmel, Jiří Dresler, Petr Pajer, Hana Kabíčková, Peter Adamík, David Modrý, Alena Myslivcová Fučíková, Jan Votýpka

**Affiliations:** ^1^Military Health Institute, Military Medical Agency, Prague, Czechia; ^2^Department of Parasitology, Faculty of Science, Charles University, Prague, Czechia; ^3^Department of Infectious Diseases, First Faculty of Medicine, Charles University and Military University Hospital Prague, Prague, Czechia; ^4^Department of Zoology, Faculty of Science, Palacký University, Olomouc, Czechia; ^5^Museum of Natural History, Olomouc, Czechia; ^6^Institute of Parasitology, Biology Centre, Czech Academy of Sciences, České Budějovice, Czechia; ^7^Department of Botany and Zoology, Faculty of Science, Masaryk University, Brno, Czechia; ^8^Department of Veterinary Sciences/CINeZ, Faculty of Agrobiology, Food and Natural Resources, Czech University of Life Sciences Prague, Prague, Czechia; ^9^Department of Biology, University of Hradec Králové, Hradec Králové, Czechia

**Keywords:** *Bartonella gliris*, *Bartonella grahamii* subsp. *shimonis*, *Bartonella* Adhesin A, cultivation-related genomic changes, gene deletion

## Abstract

Bartonelloses are neglected emerging infectious diseases caused by facultatively intracellular bacteria transmitted between vertebrate hosts by various arthropod vectors. The highest diversity of *Bartonella* species has been identified in rodents. Within this study we focused on the edible dormouse (*Glis glis*), a rodent with unique life-history traits that often enters households and whose possible role in the epidemiology of *Bartonella* infections had been previously unknown. We identified and cultivated two distinct *Bartonella* sub(species) significantly diverging from previously described species, which were characterized using growth characteristics, biochemical tests, and various molecular techniques including also proteomics. Two novel (sub)species were described: *Bartonella grahamii* subsp. *shimonis* subsp. nov. and *Bartonella gliris* sp. nov. We sequenced two individual strains per each described (sub)species. During exploratory genomic analyses comparing two genotypes ultimately belonging to the same species, both factually and most importantly even spatiotemporally, we noticed unexpectedly significant structural variation between them. We found that most of the detected structural variants could be explained either by prophage excision or integration. Based on a detailed study of one such event, we argue that prophage deletion represents the most probable explanation of the observed phenomena. Moreover, in one strain of *Bartonella grahamii* subsp. *shimonis* subsp. nov. we identified a deletion related to *Bartonella* Adhesin A, a major pathogenicity factor that modulates bacteria-host interactions. Altogether, our results suggest that even a limited number of passages induced sufficient selective pressure to promote significant changes at the level of the genome.

## 1. Introduction

Bacteria of the genus *Bartonella* are facultative intracellular pathogens that parasitize erythrocytes and endothelial cells (Han et al., 2022). They have been described in a wide spectrum of mammalian hosts including rodents, carnivores, ungulates and humans (e.g., Cheslock and Embers, 2019). Members of the genus *Bartonella* are causative agents of rather neglected emerging human infectious zoonotic diseases altogether called bartonelloses.

While infections in natural hosts are mostly asymptomatic even though chronic bacteremia is developed (Jacomo et al., 2002; Harms and Dehio, 2012), the described symptoms of bartonelloses in humans range from mild symptoms such as fever to severe ones such as encephalitis or arthritis (Okaro et al., 2017; Krügel et al., 2022). Although most of the human cases are connected primarily with only three species (*B. bacilliformis*, *B. quintana*, and *B. henselae*; Okaro et al., 2017), it has been hypothesized that virtually any *Bartonella* species can have zoonotic potential (Breitschwerdt and Kordick, 2000; Breitschwerdt et al., 2010; Okaro et al., 2017). Due to the rather difficult diagnosis, only recent studies have shown how common not only seroprevalence, but also active bacteremia can be in humans (Pitassi et al., 2015; Portillo et al., 2020). Infection of vertebrate hosts is mediated primarily through a bite or infected feces of blood-sucking arthropods such as fleas or lice (e.g., Iannino et al., 2018).

In recent years, there has been a rapid increase in the number of recognized *Bartonella* species (Breitschwerdt, 2017). Currently, there are roughly more than 100 recognized (sub)species, although some of them yet lack a formal description (Parte et al., 2020). Most of them resulted from adaptive radiation triggered by acquisition of a key molecular system that is directly related to their unique ability to colonize diverse mammalian hosts (Jacomo et al., 2002; Engel et al., 2011). Thus, individual *Bartonella* species adapted/specialized to distinct hosts which has ultimately resulted in their compelling species diversity, especially in rodents (Gutiérrez et al., 2015). The prevalence of bartonellae in various small rodents typically varies from 30% to 90% and we are not aware of any study that has reported a naive rodent population (Knap et al., 2007; Tołkacz et al., 2018; Mardosaitė-Busaitienė et al., 2019).

Within this study we focused on edible dormice (*Glis glis*), small rodents with nocturnal activity and obligatory seasonal hibernation (Kryštufek, 2010); distributed in Europe and Asia Minor (Amori et al., 2021). Edible dormice are known for their long lifespan (up to 10 years) as well as their intermittent breeding strategy, which make their life-history traits unique among rodents (Pilastro et al., 2003). Whereas in the past edible dormice were considered a delicacy and hunted or bred, now they enter households voluntarily and come into frequent contact with people, which can pose a risk of transmission of various etiological agents (Büchner et al., 2018).

The main aim of this study is to describe and characterize novel isolates of *Bartonella* (species and subspecies) cultivated from the blood of edible dormice captured in the Czech Republic. Furthermore, we describe our experience with *Bartonella* cultivation and most importantly, it’s almost immediate effects on the bacterial genomes, when only a few passages appear to be sufficient to initiate (selective) changes.

## 2. Materials and methods

### 2.1. Edible dormice capture/sampling

Edible dormice were sampled as a part of a long-term ecological project in mixed forest stands close to Dlouhá Loučka, Olomouc district, Czech Republic (49°49′ N, 17° 12′ E). Altogether, 31 adult animals were captured in August 2019 in nest boxes that they use as resting and breeding sites (for details on the study site see Gazárková and Adamík, 2016). The animals were narcotized with an intramuscular application of ketamine/medetomidine, and the blood was sampled into microtubes with EDTA from the inferior *vena cava cranialis* using a sterilized hypodermic needle. Thereafter, the dormice were aroused from narcosis using Revertor and put back into their original nest boxes. The dormice were handled on the basis of permission issued by the Regional Authority of the Olomouc Region (KUOK 61548/2017). Field study protocols were approved by the Ethical Committees of Palacky University and the Ministry of Education (1/2011, 5525/2008-30).

### 2.2. Bacteria cultivation and PCR screening

The recommendations described in Gutiérrez et al. (2017) were followed for *Bartonella* cultivation (see also Riess et al., 2008). The cultivation medium consisting of Schneider’s Insect Medium (Sigma Aldrich) supplemented with fetal calf serum (10%), saccharose (5%), and Amphotericin B (0.1%) was filtered through a 0.22 μm filter (MILEX) into a new tube before use. The medium thus prepared was added to the blood samples. Such dilution in liquid media has been shown to improve the cultivation of bartonellae by limiting the overgrowth and influence of co-infecting bacteria that are common in wild animals (Gutiérrez et al., 2017). The samples were inoculated on chocolate agar plates and cultivated at 37°C in an atmosphere enriched with 5% CO_2_ for up to 6 weeks. *Bartonella* presence was initially determined by the morphology of the colonies and a total of 32 candidate colonies per blood sample were further examined by PCR. To obtain clones of particular *Bartonella* strains, a single colony growing on the original plate was inoculated onto a new agar plate. The same process was repeated two more times and these cultures (passaged at total three times consecutively; grown four times on agar plates) were considered to be a clone of a single *Bartonella* strain. These retrieved clonal lineages were used for further analysis and characterization.

DNA extracted from selected colonies and all of the original blood samples were subjected to PCR screening of the citrate synthase gene (*gltA*, 750 bp). DNeasy Blood & Tissue Kit (QIAGEN) was used for DNA extraction according to the product manual. For detailed information about the PCR including cycling conditions and sequences of primer pairs see Majerová et al. (2021). All *Bartonella* positive PCR products were purified using the ExoSAP-IT™ (Thermo Scientific) and sequenced by the BIOCEV sequencing laboratory of the Faculty of Science, Charles University, Prague.

### 2.3. Phylogenetic analysis of amplicon sequencing data

All sequence manipulations and analyses took place in the Geneious Prime environment v2022.1.1.^1^ The amplicon sequencing data were visually controlled and adjusted, including end trimming and correction for mis-called nucleotides (e.g., Crossley et al., 2020). Preliminary taxonomic classification of such sequences was retrieved through BLASTn against the NCBI GenBank database. Next, we selected representative reference sequences for phylogenetic analysis, particularly with respect to the phylogenetic context. The phylogenetic inference was performed using PhyML (Guindon et al., 2010) and model selection was based on the jModelTest (Posada, 2008).

### 2.4. Transmission electron microscopy

One strain of each novel (sub)species was used for a morphological examination by electron microscopy. The samples were prepared by negative staining, formvar-coated carbon-reinforced grids were used, and the samples were stained with a 2% solution of phosphotungstic acid. In addition, fixation and embedding were also used as another method of sample preparation for electron microscopy. The samples were fixated in the solution of 2.5% glutaraldehyde (Polysciences) and 5 mM CaCl_2_ in 0.1 M cacodylate buffer (pH 7.2), dehydrated and subsequently embedded in Epon-Durcupan. The embedded samples were cut into ultrathin sections, placed on a copper grid, and contrasted with lead citrate and uranyl acetate. The samples prepared by embedding and negative staining were visualized using a transmission electron microscope (JEM 200CX Jeol). The length and width of the bacteria were measured, mean estimates of length and width were calculated.

### 2.5. Biochemical characterization

Biochemical characterization was performed on one-week old cultures of the type strains of the novel (sub)species and the reference strain of *B. henselae* CNCTC 5656 (serving as a control), provided by the National Institute of Public Health (Czech Republic). For each strain, several colonies from a clonal lineage were resuspended in sterile distilled water and transferred into the standard commercial biochemical panel (API 20 E; bioMérieux, Marcy l’Etoile, Lyon, France) that was used according to the manufacturer’s instructions (incubation at 37°C for 24 h). The results were interpreted using the API reading scales according to the manufacturer’s directions. Catalase activity was tested by immersion of a bacteria in 3% H_2_O_2_. Catalase-positivity was assessed by the appearance of oxygen bubbles.

### 2.6. Library preparation and WGS sequencing

Based on results from capillary sequencing of several genes, two possibly novel (sub)species were selected for Whole Genome Sequencing (WGS) and subsequent analyses, each represented by two strains/genotypes (i.e., biological replicates).

Libraries for Illumina sequencing were prepared using the Nextera XT DNA Library Preparation Kit. DNA concentrations were measured by a Qubit™ fluorometer (Invitrogene) device in combination with a Qubit™ dsDNA HS Assay Kit (Invitrogene). Library length profiles were measured using the Qsep 1 Capillary Electrophoresis System (BiOptic). Prepared libraries were sequenced on the Illumina iSeq 100 Sequencing System (2 × 150 paired-end).

Libraries for Oxford Nanopore Technologies (ONT) sequencing were prepared from a high molecular weight DNA using a Ligation Sequencing Kit (SQK-LSK109) and Native Barcoding Expansion 1–12 and 13–24 kits (EXP-NBD104 and EXP-NBD114). Libraries were sequenced on the ONT GridION platform using the R9.4 chemistry (Flow-Cell). Sequencing data were base-called, i.e., transmission from physical changes in the electric current signal measured by the ONT sequencing device to biologically relevant bases, using Guppy v5.1.13 (e.g., Wick et al., 2019).

### 2.7. Genome assembly and annotation

Basic characteristics of the Illumina libraries were checked using the FastQC tool (Andrews, 2010). Considering all four samples, combined coverage both from Illumina as well as from the ONT sequencing reached values >>100× (see Supplementary Table 1), which shall be sufficient for assembly of a rather small bacterial genome. Genomes were assembled using the MaSuRCA v4.0.9 hybrid assembler, which can utilize both the long error-prone reads as well as short accurate Illumina reads data in parallel (Zimin et al., 2017). Gene annotation was originally performed using Prokka v1.14.6 (Seemann, 2014) and finally by the NCBI Proteomic Genome Annotation Pipeline v2022-10-03 (Tatusova et al., 2016). The resulting genome sequences were deposited into NCBI GenBank repositories.

### 2.8. Phylogenomic analyses of WGS data

We deliberately selected representative sequences across bartonellae to set our samples into the phylogenetic context (see Supplementary Table 2). For selected species/strains we downloaded protein sequences from NCBI databases. We utilized OrthoFinder v2.5.4 to identify single copy orthologous genes present in all samples (Emms and Kelly, 2019). Next, Multiple Sequence Alignments were conducted in the software MAFFT v7.505 (Katoh and Standley, 2013). In order to estimate the species tree, we concatenated individual genes using the AMAS tool (Borowiec, 2016). Nevertheless, beyond the species tree itself, all individual Maximum Likelihood gene trees were reconstructed by IQ-TREE v2.2.0 (Nguyen et al., 2015) using the extended model selection with a free rate of heterogeneity in combination with 1,000 ultrafast bootstrap replicates (Nguyen et al., 2015; Kalyaanamoorthy et al., 2017; Hoang et al., 2018). Finally, both gene Concordance Factors (gCF) and site Concordance Factors (sCF) were evaluated (Minh et al., 2020). The resulting tree was visualized using iTOL v5 (Letunic and Bork, 2021).

### 2.9. Pan-genomic analyses

The average nucleotide identity, i.e., overall sequence similarity, of the novel strains to their nearest relatives was estimated from the genomic sequences using the OrthoANI tool (Lee et al., 2016).

Further, we utilized the R package micropan designed for microbial Pan-genome analyses and visualizations (Snipen and Liland, 2015; R Core Team, 2021). However, instead of relying on Prodigal annotations (Hyatt et al., 2010) as suggested by the micropan pipeline, we utilized original NCBI and Prokka annotations, which seemed to provide less problematic/ambiguous results. All genes of all species were clustered to gene families into a so-called pan-matrix. This allowed us to visualize the relation between pan-genome and core-genome. Next, we estimated whether the pan-genome is open or closed based on a Heaps law model (Tettelin et al., 2008). Further, we computed the principal component analysis (PCA) using the pan-matrix, which clustered individual species based on similarity of their gene contents (presence/absence).

### 2.10. Comparative genomic analyses—structural variation

Given that we have for each (sub)species sequenced two independent strains, it allowed us to assess their intra-specific variability. The overall similarity of the strains to each other was estimated by the OrthoANI tool and further assessed by the phylogenomic approach. We mapped ONT long reads from one strain to the other using the rather restrictive mapper Winnowmap2 (Jain et al., 2022). Structural Variations (long deletions) were estimated using Sniffles v2.0.7 (Sedlazeck et al., 2018). Similarly, we inferred such events from Illumina data using the pipeline consisting of BWA-MEM v0.7.17 (Li, 2013), gammaBOriS (Sperlea et al., 2020) and CNV-BAC (Wu et al., 2020). The detected events were controlled visually using the Integrative Genomics Viewer (Robinson et al., 2011). Only substantial events of size > 1 kb were considered.

Furthermore, we screened the genomes for prophages using the Prophage Hunter web-tool (Song et al., 2019). The genome collinearity was assessed using the Gepard tool (Krumsiek et al., 2007). The presence of coiled-coil segments within selected proteins was assessed using the Quick2D online-tool (Delorenzi and Speed, 2002; Gabler et al., 2020). We utilized GenomeTools to visualize annotation features (Gremme et al., 2013).

### 2.11. Proteomics and mass spectrometry

We provide a brief summary of the methods used here; detailed information about the methods may be found in the Supplementary material. Samples of both novel (sub)species, cultivated on agar plates as described above, were analyzed by proteomic techniques. Several colonies per each sample were harvested and lysed for the purposes of protein extraction and digestion. Isolated peptides were subject to the shotgun Label Free Quantification (LFQ) proteomic analysis. All samples were processed using the UltiMate 3,000 RS Liquid Chromatography Nano System Flow Meter with the Orbitrap Mass Spectrometer (Thermo Scientific) and the acquired data were evaluated using MaxQuant software (Cox and Mann, 2008).

## 3. Results

### 3.1. *Bartonella* prevalence in edible dormice

*Bartonella* cultivation was successful for 23 out of the 31 edible dormice individuals captured in August 2019 (for one animal not enough blood was taken and only DNA isolation was performed, three animals were *Bartonella*-negative and in four samples other bacterial contamination prevented *Bartonella* positivity assessment by the cultivation method). Two different *Bartonella* (sub)species were identified: in 14 dormice specimens, infection by a single *Bartonella* (sub)species was observed whereas in nine of them, colonies of the two *Bartonella* (sub)species occurred alongside one another.

However, PCR analysis of the original host blood samples revealed an even higher positivity (30/31; 96.8%) and proportion of mixed infections by the two *Bartonella* (sub)species (20/30; 66.7%).

### 3.2. Bacteria cultivation

Several *Bartonella* clonal lineages were acquired during the cultivation. We selected two clonal lineages per each presumably novel (sub)species which were further extensively analyzed. Specifically, the strains denoted as GG3s1 and GG23s2 represented *B. grahamii* subsp. *shimonis* subsp. nov., whereas the strains denoted as GG20g1 and GG6g2 represented *B. gliris* sp. nov.

Considering all of the isolated strains, optimal colony growth was observed at 37°C with 5% of CO_2_, i.e., the conditions commonly used for *Bartonella* species cultivation (Brenner et al., 1993; Gutiérrez et al., 2017). Any other experimental conditions led to slower or mostly suppressed growth (see Supplementary Figure 1). We did not observe any considerable differences in growth rates between the studied isolates.

The morphology of the colonies did not systematically differ between the individual strains or even individual (sub)species. Instead, we noticed some variability common to all of them. Overall, the colonies were round with cleanly cut edges of white to yellow color, ranging from 0.6 to 3 mm in diameter. The colonies differed in their consistency from hard colonies attached to the surface to creamy ones without an attachment. The morphology of the colonies is comparable to that of other *Bartonella* species (Molia et al., 2016; Gutiérrez et al., 2020).

### 3.3. Transmission electron microscopy

Transmission electron microscopy revealed rod-shaped bacilli without any flagella or other noticeable structures (see Supplementary Figure 2). The size of *Bartonella grahamii* subsp. *shimonis* subsp. nov. ranged in length from 0.83 to 1.43 μm (mean = 1.1 μm, SD = 0.06 μm) and the width from 0.26 to 0.41 μm (mean = 0.34 μm, SD = 0.03 μm). The size of *Bartonella gliris* sp. nov. ranged in length from 0.76 to 1.26 μm (mean = 1 μm, SD = 0.14 μm) and the width from 0.3 to 0.51 μm (mean = 0.38 μm, SD = 0.06 μm). In general, the strains in the study fell within the expected size range and did not possess any apparent morphological traits that would differentiate them from other related *Bartonella* species (Breitschwerdt and Kordick, 2000; Minnick and Anderson, 2015).

### 3.4. Biochemical characterization

All three tested *Bartonella* species (each represented by a single strain/genotype) were negative for the catalase activity test and all of the tests included in the commercial API 20E panel, except for production of the enzyme gelatinase, which was positive for *Bartonella gliris* sp. nov. and *B. henselae*.

The results for the reference strain *B. grahamii* subsp. *shimonis* subsp. nov. partially contradict former reports of various type strains of *B. grahamii* for which, for example, Voger-Proskauer test activity has been reported (Birtles et al., 1995). However, they are in agreement with other reports of *Bartonella* spp. (do Amaral et al., 2022). In general, standard biochemical tests can not characterize or even distinguish members of the genus *Bartonella* (Diddi et al., 2013; Celebi et al., 2021), and thus no further tests were performed.

### 3.5. Phylogenetic/phylogenomic analyses

**Figure 1:**
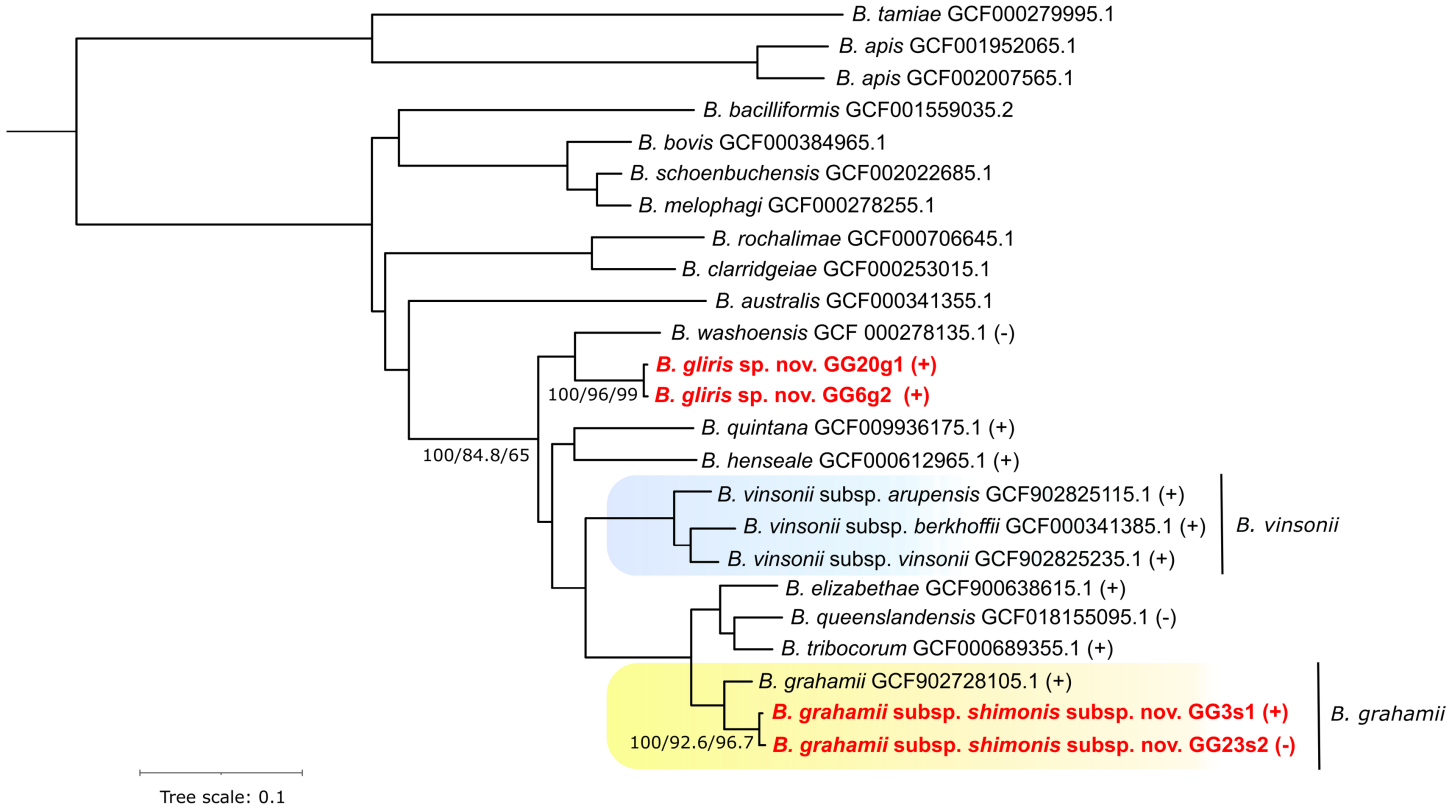
Phylogenomic species tree of *Bartonella* species/strains estimated from 501 single copy orthologous protein sequences. Branch supports expressed as Bootstrap values, gene Concordance Factors and site Concordance Factors are provided for selected branches. Symbols (+) and (−) indicate “*Wolbachi*a endosymbiont wVitA of *Nasonia vitripennis* phage WOVitA1” prophage presence or absence, respectively, within a given species or strain. Newly described strains are highlighted by red font color. Background color indicates species-subspecies clusters.

Already the primary phylogenetic analyses revealed that the strains under study are possibly novel, i.e., they formed separate clusters in the inferred phylogenies (see Supplementary Figure 3). In particular, one (sub)species clustered with the *B. grahamii* species group whereas the other appeared as a sister lineage to *B. washoensis*. These results were comparable with the phylogenomic approach that utilized sequence data from 501 genes (see Figure 1). The branches separating the analyzed (sub)species from their nearest relatives are well supported by the Concordance Factors metrics, both gCF and sCF, which also applies to the most branches of the entire phylogeny.

The average nucleotide identity (ANI) scores estimated from the genomic sequences for the isolates GG3s1 and GG23s2 related to *B. grahamii* were 93.6% and 93.7%, respectively. The ANI estimates for the isolates GG3s1 and GG23s2 that appeared as sister lineage to *B. washoensis* only reached values of 88.6% and 88.9%, respectively (all estimates are based minimally on comparison of contigs > 1.45Mbp). The ANI values estimated between each of these strains and the rest of the entire phylogeny can be found in Supplementary Table 3. Basic statistics of the assembled genomes may be found in Supplementary Table 4.

### 3.6. Pan-genome analyses

**Figure 2:**
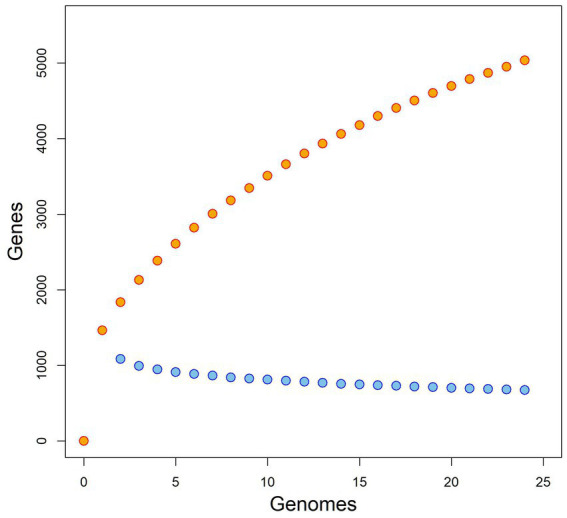
Pangenome and core-genome size for *Bartonella* spp., rarefaction “curves” plotted in orange and blue color, respectively.

**Figure 3:**
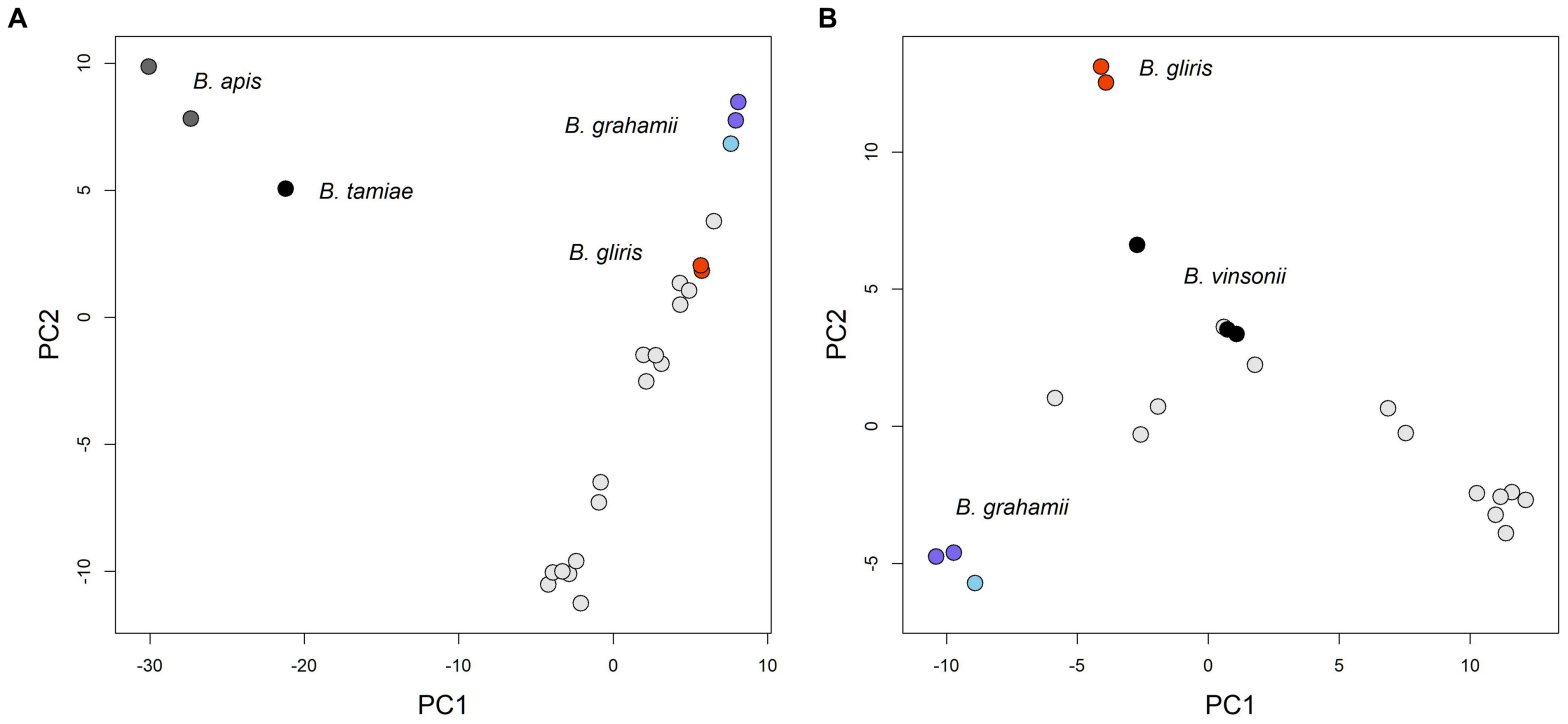
PCA figures based on similarity of gene contents between individual strains (gene presence/absence). Only several species could be highlighted both by distinct color and caption in the figures. Within the *Bartonella grahamii* group, *B. grahamii* subsp. *shimonis* subsp. nov. samples are depicted by darker blue. **(A)** The phylogenetically most distant species, i.e., *B. apis* and *B. tamiae*, appeared as clear outliers compared to the rest of samples in this analysis and factually dominated the Principal Component 1 (PC1); PC1 explained 28.0% of the total variability and PC2 13.0%. **(B)** We excluded *B. apis* and *B. tamiae*, which resulted in finer separation of the rest of the samples; PC1 explained 23.0% of the total variability and PC2 10.4%.

Analysis of the pangenome showed that the core genome is relatively small, consisting of about 674 genes, while the pangenome is relatively large, probably exceeding more than 5,036 genes (see Figure 2). Based both on the shape of the pan-genome graph and the calculation according to Tettelin et al. (2008) (alpha estimate = 0.582), the pan-genome appears to be open. The PCA analysis that was based upon presence/absence of individual genes, contrary to phylogenetic estimates that are virtually based on sequence (dis)similarity, to some extent reflected patterns of the phylogenomic inference/history. In particular, the phylogenetically most distant (outgroup) species, i.e., *B. apis* and *B. tamiae*, are clearly separated from the rest of the samples (see Figure 3). In fact, the difference of those species was so significant that it hampered any comparisons within the ingroup species/samples, and therefore we excluded them from the analysis.

### 3.7. Comparative genomic analyses

The overall similarity between both strain pairs expressed as Average Nucleotide Identity was 99.18% for *B. grahamii* subsp. *shimonis* subsp. nov. and 99.28% for *B. gliris* sp. nov. isolates. Furthermore, genome collinearity is almost fully preserved in both cases (see Supplementary Figures 4, 5), with the sole exception of deletion events (ranging in size typically from ~20 to ~55 kb).

Except for one such deletion event, which will be further described in detail, virtually all of the substantial deletions are linked with prophages. Moreover, such prophages are typically predicted with a high degree of certainty and denoted as “Active.” This suggests that the prophages most likely maintained their ability to excise from the genome (under certain conditions). As an example, we can describe probable loss of “*Wolbachia* endosymbiont wVitA of *Nasonia vitripennis* phage WOVitA1” in the *B. grahamii* subsp. *shimonis* subsp. nov. GG23s2 strain. Interestingly, this prophage contains a gene for Lysin *sensu* (Low et al., 2011), whose role in a bacteria/prophage is speculative. On one hand, it might serve the prophage leaving the cell by lysis of the cell wall (Fischetti, 2008), on the other hand, the presence of prophage Lysin affects the phenotype of a bacteria (Aucouturier et al., 2018). The presence of this prophage seems to be typical within the considered part of *Bartonella* phylogeny (see Figure 1). The only species in which the prophage was not detected were *Bartonella washoensis* and *Bartonella quintana*. The latter one has further a quite small (reduced) genome size (~1.6 Mbp) compared to a typical *Bartonella* species (~2 Mbp).

The prophage locus is bounded on both sides by an identical short sequence motif (“TCCCTCTCTCTCCGCCAT”). Whereas in the strain where the prophage was presumably excised, only a single copy of this motif was found. This suggests that the phage excision was probably achieved through site-specific tyrosine recombinase/integrase that is actually encoded by the prophage (e.g., Van Houdt et al., 2012).

### 3.8. *Bartonella* Adhesin A

The presumable deletion event that cannot be evidenced as prophage excision, also occurred in the *B. grahamii* subsp. *shimonis* subsp. nov. GG23s2 strain, moreover, in close proximity to the described prophage excision site. Interestingly, this ~22 kb long sequence contained *Bartonella* Adhesin A (BadA) *sensu* (Thibau et al., 2022), i.e., ~4,000 amino acids long protein coding gene with defined internal repetitive motifs. Moreover, the locus contained several accessory BadA subunits (or remnants). BadA is known to represent a key virulence factor (Thibau et al., 2022).

**Figure 4:**
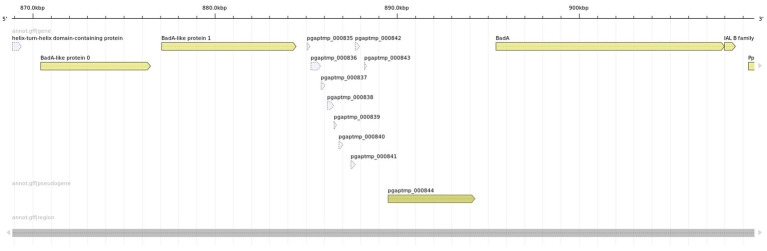
Presumable wild type *Bartonella* Adhesin A (BadA) locus identified in *Bartonella grahamii* subsp. *shimonis* GG3s1 strain. The locus contains three BadA(-like) genes that are well diversified at the sequence level and structural properties. The rightmost copy represents the BadA *sensu* (Thibau et al., 2022), i.e., the presumed functional copy of the *Bartonella* Adhesin A with described structure and properties.

The lost copy of BadA clearly represents an ortholog of BadA *sensu* (Thibau et al., 2022), but beyond that, two other copies of BadA-like sequences were identified (see Figure 4). BadA-like protein 1 is sequentially related to the BadA gene, but differs in length and structure. It is substantially shorter and does not contain the typical repetitive motifs (coiled coils). BadA-like protein 1 could be due to its structural properties as well as topological position considered to be orthologous to a sequence described as BadA pseudogene by Thibau et al. (2022). BadA-like protein 0 differs considerably sequentially from the two other sequences.

**Figure 5:**
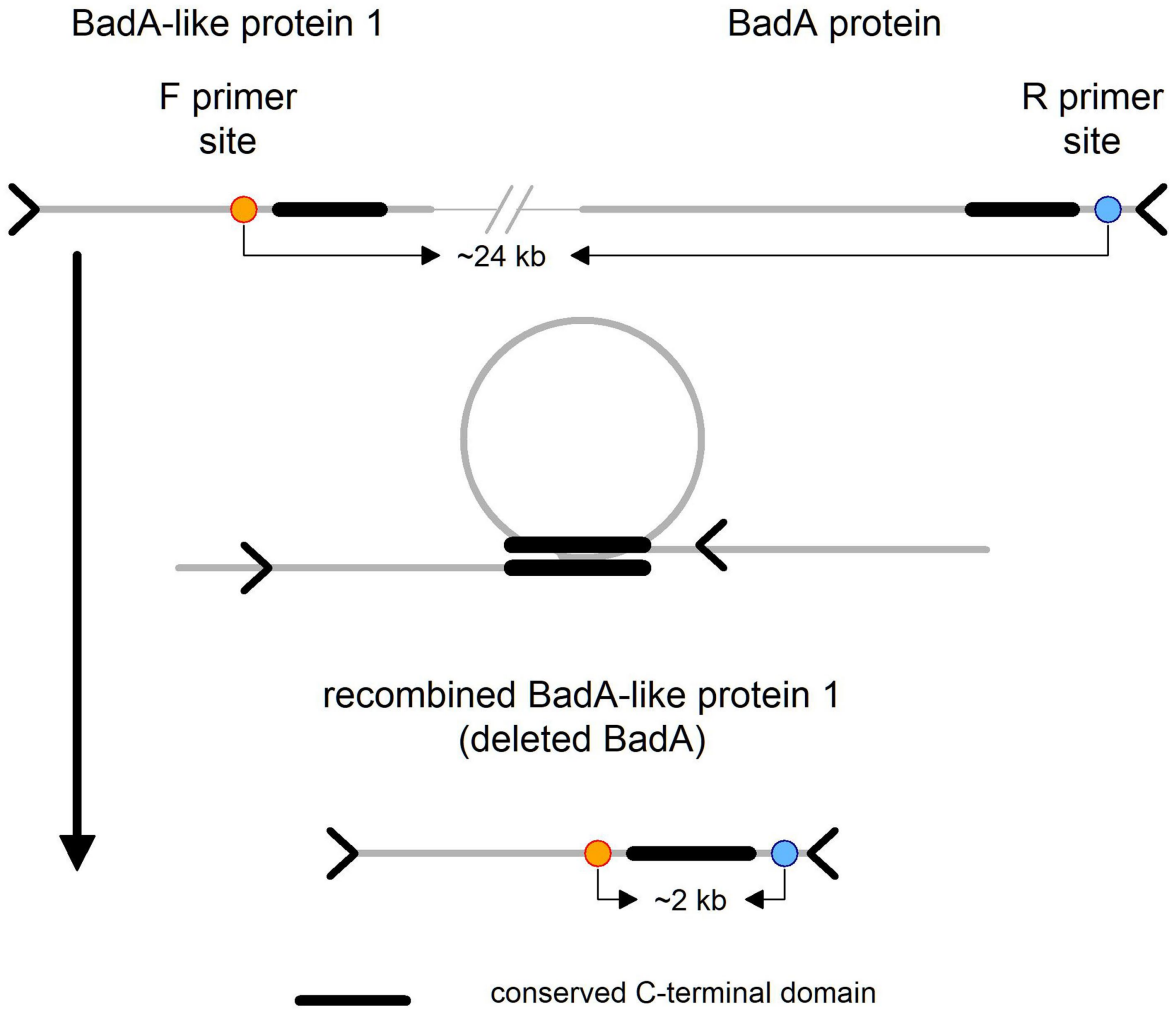
Schematic representation of BadA-like 1 and BadA proteins. On the top of the figure is illustrated the wild type locus variant with primer sites, forward on the left and reverse on the right side. In the middle is depicted the probable cause of the deletion event, i.e., recombination between the conserved C-terminal domains, which has resulted in the recombined (“chimeric”) variant of BadA-like protein 1 depicted at the bottom of the figure.

Further, we revealed the mechanistic basis of BadA locus deletion. BadA and BadA-like protein 1 are in general quite divergent (63% identity), however they share perfect homology at a part of the C-terminal domain (~460 aa). We noticed that BadA-like protein 1 in the genome with the deleted BadA locus is “chimeric,” i.e., its C-terminal domain comes from the BadA. This suggests that the BadA locus was excised through recombination between the conserved C-terminal domains (see Figure 5).

### 3.9. PCR detection of BadA-deficient phenotype within host blood samples

The architecture of the BadA locus itself is complicated and most importantly repetitive. This fact has complicated the development of a simple PCR test that could theoretically prove the presence/absence of the BadA deleted phenotype within the host. We designed primers that should yield a product of size ~2 kb in the deletion phenotype and no product in the wild type variant (primers are ~24 kb apart). Further, we developed *B. grahamii* subsp. *shimonis* subsp. nov. specific primers that should yield a product of similar size as a positive control. We optimized and confirmed the function of the primers using high-quality bacterial DNA (also utilized for sequencing). Surprisingly, we observed that even the presumably wild type strain of *B. grahamii* subsp. *shimonis* subsp. nov. (GG20g1) contains the deletion phenotype, however its frequency was several fold lower compared to the wild type variant. This observation is based on the fact that while the deletion phenotype was undetectable in the sequencing data, the PCR analysis weakly detected it (see Supplementary Figure 6).

Finally, we used such primers on the DNA extracted directly from the blood of two dormouse specimens from which the strains were originally isolated (and cultivated). The PCR reaction required different conditions (more cycles) compared to pure bacterial DNA (detailed information including primer sequences is provided in the Supplementary material). Nevertheless, in both cases we clearly detected the positive control PCR product, but we did not reliably detect the deletion phenotype marker (see Supplementary Figure 6). However, in one of the host samples, the results of this assay were inconclusive because we weakly detected the product at about the size of the expected marker. But there were also other products that were not detected in any other reaction.

Therefore, we sequenced the products in order to definitively prove or disprove the presence of the deletion marker in the original host blood samples (ONT GridION platform). While in the positive control we got mostly reads with length corresponding to the given marker (~1,970 bp), in the questionable sample we got a mixture of reads without obvious peak and also without any clear identification (estimated by BLASTn; data not shown). We can only speculate that these are probably repetitive sequences from the host genome, but we certainly did not detect the expected marker.

### 3.10. Proteomics and mass spectrometry

Using the Label Free Quantification (LFQ) proteomic approach, a total of 523 quantifiable proteins were detected. The observed quantities of the proteins are expressed as log2 transformation of LFQ intensities (Cox et al., 2014). Values ranging from 19 to 34 reflect the dynamic range of the mass spectrometry-based workflow. LFQ intensities below this value were considered as non-analyzable by the implemented qualitative test (Cox et al., 2011). We focused especially on analysis of *Bartonella* Adhesin A(-like) proteins. But since these proteins are similar to some extent, we considered only peptides that could be unambiguously mapped exclusively to a single gene. While the proteomic evidence for expression of the BadA protein is rather weak, i.e., based on a single peptide (“VEGDSLVKQDK”), the expression of BadA-like protein 1 was evident (see Supplementary Figure 7). We did not detect any peptide originating from BadA-like protein 0.

### 3.11. Description of novel *Bartonella* (sub)species

#### 3.11.1. *Bartonella grahamii* subsp. *shimonis* subsp. nov. (sĭmŏnis, in honor of Shimon Harrus from the Hebrew University of Jerusalem, Koret School of Veterinary Medicine, for his extensive contribution to the study of the genus *Bartonella*)

The cells are rod-shaped bacilli without any additional structure with a length of 0.8 to 1.4 μm and a width of 0.3 to 0.4 μm. Within 10 days on chocolate agar plates under optimal conditions (37°C with 5% CO_2_), the white-yellow colonies appear round and vary in size (0.6 to 3 mm). The type strain GG3s1 was negative for b-galactosidase (ONPG hydrolysis), decarboxylation of arginine, lysine, and ornithine, utilization of citrate, hydrogen sulfide production, urease, tryptophan deaminase, indole production from tryptophan, production of the enzyme gelatinase, Voges–Proskauer reactions, catalase activity, and fermentation of glucose, mannose, inositol, sorbitol, rhamnose, sucrose, melibiose, amygdalin, and arabinose. The type strain carried a single circular chromosome with a size of 2.26 Mb and GC content of 37.92%. The closest species with a published genome is *B. grahamii* (93.6% ANI). The type strain GG3s1, isolated from the blood of edible dormice (*Glis glis*) sampled in the Olomouc district of the Czech Republic, was deposited in the Department of Parasitology, Charles University, Prague, and in the Military Health Institute, Prague (sample ID #000522).

#### 3.11.2. *Bartonella gliris* sp. nov. [glīris, the name refers to the only known host of this bacteria, the edible dormouse (*Glis glis*)]

The main phenotypic characteristics are identical to those of the genus *Bartonella*. Within 10 days on chocolate agar plates under optimal conditions (37°C with 5% CO_2_), the white-yellow colonies appear round and vary in size (0.6 to 3 mm). The cells are rod-shaped bacilli without any additional structure with a length of 0.8 to 1.3 μm and a width of 0.3 to 0.5 μm. The type strain GG6g2 is positive for the enzyme gelatinase and negative for b-galactosidase (ONPG hydrolysis), decarboxylation of arginine, lysine, and ornithine, utilization of citrate, hydrogen sulfide production, urease, tryptophan deaminase, indole production from tryptophan, Voges–Proskauer reactions, catalase activity, and fermentation of glucose, mannose, inositol, sorbitol, rhamnose, sucrose, melibiose, amygdalin, and arabinose. The type strain carried a single circular chromosome a size of 1.95 Mb and GC content of 39.26%. The closest species with a published genome is *B. washoensis* (88.9% ANI). The type strain GG6g2, isolated from the blood of edible dormice (*Glis glis*) sampled in the Olomouc district of the Czech Republic, was deposited in the Department of Parasitology, Charles University, Prague, and in the Military Health Institute, Prague (sample ID #000521).

## 4. Discussion

### 4.1. Species recognition and delimitation in microbiology

Species recognition is somewhat tricky in bacteria. In recent history, even the very existence of bacterial species was questioned, mainly due to the exaggerated importance of a horizontal gene transfer (HGT) that could melt the presumed species boundaries (Riley and Lizotte-Waniewski, 2009). Species concept and its definition in microbiology remains to be problematic till now (Bobay, 2020). Instead, so-called Core Genome Hypothesis (CGH) was proposed to solve the apparent paradox of HGT and rapid gene flow in general on one hand and the objective existence of distinct bacterial species on the other hand (Riley and Lizotte-Waniewski, 2009). Basically, the CGH defines a stable part of a genome (core), which is responsible for a maintaining of the species identity, whereas the rest of the genome allows for rapid adaptation to an ever-changing environment. Thus far, only CGH offers a reasonable perspective on the fact that analysis of over 2,000 genomes of *E. coli* resulted in a pan-genome size reaching 90,000 genes (Land et al., 2015; Iranzadeh and Mulder, 2019).

Nowadays, the standard for species delimitation is represented by a rather pragmatic threshold of average nucleotide identity (ANI) estimated from genomic sequences (Bobay, 2020). As a rule of thumb, if a strain shows ANI to any nearest described species lower than 95%, it might be considered a novel species (Chan et al., 2012; Bobay, 2020). So, the values estimated in the analyzed strains (88.9% and 93.7%), demonstrated that they represent well established and independent evolutionary entities. Nevertheless, even the threshold of 95% does not apply to current phylogenies categorically, which might be explained by historic or other reasons (Welch et al., 1999). Particularly, many species/taxa descriptions come from the times when genome sequencing was not as common as it is today and to obtain exact estimates of species divergence was complicated (e.g., Brenner et al., 1982; Richter and Rosselló-Móra, 2009) and/or the authors may have had other reasons for deciding not to adopt the species rank (e.g., Kordick et al., 1996; Welch et al., 1999). If we estimate the ANI values of *B. vinsonii* subsp. *berkhoffii* and *B. vinsonii* subsp. *arupensis* to *B. vinsonii* (and even between each other) today, they fall significantly below the 95% threshold, 94 and 93%, respectively. This observation was recently acknowledged by do Amaral et al. (2022), who indeed suggested taxonomic reclassification of the genus *Bartonella*. In summary, we also considered other criteria beyond the ANI estimates.

### 4.2. Defining novel (sub)species

Specifically, we considered the results of the pangenome analysis, which suggested that while the core genome of *Bartonella* is rather small, the pangenome appears to be open. This characteristic does not seem to be of major importance considering the above-mentioned study of *E. coli* pan-genome (Land et al., 2015), however, not all bacteria share this feature (Hemsley et al., 2019). Both HGT as well as gene loss and duplication were described to play a role in *Bartonella* genome dynamics (Québatte and Dehio, 2019; Banerjee et al., 2020). The PCA results showed two important things. First, that *Bartonella gliris* sp. nov., seems to represent a unique genome composition that is not directly related to any other species. And second, that *Bartonella grahamii* subsp. *shimonis* subsp. nov. closely clustered together with *B. grahamii*. Moreover, even after inclusion of this new subspecies, the *B. grahamii* (sub)species would still form a compact and distinct cluster, especially when compared with the *B. vinsonii* (sub)species.

Furthermore, based on long-term monitoring of the prevalence of *Bartonella* infections in small mammals carried out in the Czech Republic (Obiegala et al., 2019; Majerová et al., 2021), we noticed an important biological difference between the described (sub)species. *Bartonella grahamii* subsp. *shimonis* subsp. nov. displays rather low host specificity and is be found in a broad range of mammalian hosts similarly to *B. grahamii* (Berglund et al., 2010). Within the study of Majerová et al. (2021) the newly described *Bartonella grahamii* subsp. *shimonis* subsp. nov. was already detected in hedgehogs (*Erinaceus europaeus* and *E. roumanicus*) and squirrels (*Sciurus vulgaris*), based upon PCR screening of the *gltA* and *rpoB* genes, and the detected genotype was provisionally designated as *Bartonella* sp. SCIER (see Supplementary Figure 3). On the other hand, *Bartonella gliris* sp. nov. has been detected solely in edible dormice to date; nevertheless, further investigations would be needed to confirm this observation. But if *Bartonella gliris* sp. nov. has truly adapted to a single host, then it would represent a potentially interesting example of specific host adaptation (Withenshaw et al., 2016; Québatte and Dehio, 2019).

### 4.3. Hints of stress-induced genomic changes

In the past, the role of prophages and the effects of their residence within bacterial genomes were in the past considered simply as parasitism (Bondy-Denomy and Davidson, 2014). Nowadays, it is clear that this point of view was an oversimplification and in reality prophages might be beneficial for their bacterial host (Wang et al., 2010; Bobay et al., 2014). Despite the fact that we were not able to experimentally prove a direct causal progression of individual deletion/excision events (due to technical reasons/limitations), we think that the presented evidence strongly supports our interpretation, which furthermore represents the most parsimonious explanation of the observed phenomena.

Further, genome streamlining was recently described as a nearly universal response to naturally occurring environmental stress conditions (Simonsen, 2022). On the other hand, the study of Simonsen (2022) did not consider the role of phages/prophages, which seem to play a dominant role in our case. Actually, it has been shown that gene acquisition through prophage integration is a major evolutionary route for facultative bacterial pathogens (Busby et al., 2013). Greenrod et al. (2022) emphasized not only the role of prophages as carriers of auxiliary genes with a potential role in bacteria metabolism and gene regulation. But most importantly, they demonstrated that host evolution is virtually mirrored by phage evolution in certain cases, a phenomenon which could have resulted (only) from a shared coevolutionary history. In other words, phage possession might represent a typical feature of a certain bacterial clade, as we evidenced for the “*Wolbachia* endosymbiont wVitA of *Nasonia vitripennis* phage WOVitA1.” Therefore, we interpret its absence in one of the analyzed *B. grahamii* subsp. *shimonis* subsp. nov. strains as an excision event.

Similarly, the absence of *Bartonella* Adhesin A (BadA) locus in *Bartonella grahamii* subsp. *shimonis* subsp. nov. strain GG23s2 is considered a deletion event. BadA is known to represent a key virulence factor which mediates bacteria-host interactions and might even contribute to host immune evasion (Thibau et al., 2022). This adhesin was also identified as a key factor for adherence to host cells and biofilm formation (Müller et al., 2011; Okaro et al., 2019). Adhesins are generally a (hyper)variable class of proteins. Their variability stems from never-ending interactions between the pathogen and an adaptive host immune system (May and Brown, 2011). Their sequence as well as topological variability has already been documented in *Bartonella henselae* strains (Thibau et al., 2022). However, the presumable deletion of the ~22 kb long fragment is far beyond the scope of what Thibau et al. (2022) describe as variation and adaptation. Such an irreversible loss of the genetic repertoire can hardly be considered adaptive. Instead, it is in line with previous studies which have reported that “extensive passaging” of *Bartonella henselae* strains led to deletion of ~10 kb of the BadA locus (Riess et al., 2004). Long-term survival and proliferation of the bacteria within a host without the BadA machinery is improbable and therefore, its irreversible loss provides the strongest evidence for cultivation-induced genomic changes. This interpretation is also clearly supported by the results of PCR analysis of host blood, where we did not detect the deletion phenotype, conversely to both agar plates cultivated *Bartonella grahamii* subsp. *shimonis* subsp. nov. strains. This result on the other hand cannot exclude the possibility of natural occurrence of the deletion phenotype, but it proves that the wild type variant is dominant within a host.

The observed phenomenon could be to some extent compared to the cultivation induced genomic changes of *Coxiella burnetii* Nine Mile strain, i.e., another facultative intracellular pathogenic bacteria. For which it has been repeatedly demonstrated that cultivation ultimately leads to loss (deletion) of a defined section of the genome (Hoover et al., 2002). Moreover, this systematic deletion of several genes significantly negatively affects the pathogenicity of concerned strains compared to the wild-type (Kersh et al., 2011).

Using the proteomic approach, we demonstrated that BadA-like protein 1 was expressed in both *B. grahamii* subsp. *shimonis* sp. nov. strains and therefore cannot be considered as a pseudogene as suggested by Thibau et al. (2022). Instead, it is possible that under natural conditions some BadA variants might be preferred under specific conditions connected with the life cycle of the bacteria, i.e., vector-host dynamics.

## 5. Conclusion

Due to the frequent close contact of dormice with humans, the newly detected and described *Bartonella* (sub)species may pose a risk of zoonotic infection. Therefore, more attention should be paid to these species and their vectors.

The presumed deletion events, especially the deletion of *Bartonella* Adhesin A (BadA) locus, described in this study suggest that even relatively short-term laboratory cultivation with a low number of passages seems to represent sufficient selective force. In other words, it seems to induce enough stress to promote substantial changes at the level of the genome. Although we broadly focused on the BadA deletion within this study, it is worth to note that the importance of (presumed) prophage excision events should not be overlooked. However, in contrast to well-described genes, the significance and implications of these phenomena are very difficult to interpret.

We emphasize that a novel model that would allow us to study *Bartonella* spp. in their natural form and conditions needs to be established to confirm the findings presented in this study. Also, the role of individual *Bartonella* Adhesin A-like genes should be reviewed as we have clearly demonstrated that they are expressed during cultivation. Nevertheless, further research would be needed to reveal their possible role and function under natural conditions.

## Data availability statement

The raw sequencing data generated for this study can be found in the NCBI SRA archives. Similarly, the genome sequence data as well as the annotations may be accessed in the NCBI GenBank archives. All the data may be found under the NCBI BioProject PRJNA909980. The proteomic datasets generated and analyzed during the current study have been deposited in the ProteomeXchange Consortium via the PRIDE (Perez-Riverol et al., 2019) partner repository with the dataset identifier PXD046060 (https://www.ebi.ac.uk/pride/archive/projects/PXD046060).

## Ethics statement

The animal study was approved by Ethical Committees of Palacky University and the Ministry of Education. The study was conducted in accordance with the local legislation and institutional requirements.

## Author contributions

OB: Conceptualization, Data curation, Formal analysis, Investigation, Methodology, Supervision, Visualization, Writing – original draft, Writing – review & editing. BK: Investigation, Methodology, Writing – original draft. KV: Investigation, Methodology, Visualization, Writing – review & editing, Writing – original draft. MC: Data curation, Investigation, Methodology, Writing – original draft. JD: Funding acquisition, Investigation, Methodology, Writing – original draft. PP: Funding acquisition, Investigation, Writing – original draft. HK: Investigation, Methodology, Writing – original draft. PA: Investigation, Resources, Writing – original draft. DM: Investigation, Resources, Writing – original draft. AF: Investigation, Writing – original draft. JV: Conceptualization, Formal analysis, Investigation, Supervision, Writing – review & editing.

## Footnotes

^1^ https://www.geneious.com
