# Supplementary material for: Two novel Bartonella (sub)species isolated from edible dormice (Glis glis): hints of cultivation stress-induced genomic changes
Source: Front Microbiol. 2023 Nov 15;14:1289671. doi: 10.3389/fmicb.2023.1289671 (PMC10684924; doi:10.3389/fmicb.2023.1289671)
Supplement: Supplementary file 1 [file Data_Sheet_1.docx]

Supplementary Material

Two novel *Bartonella* (sub)species isolated from edible dormice
(*Glis glis*):
Hints of cultivation stress-induced genomic changes

Oldřich Bartoš**†*,** Běla Klimešová**†**, Karolina Volfová, Martin Chmel, Jiří Dresler, Petr Pajer, Hana Kabíčková, Peter Adamík, David Modrý, Alena Myslivcová Fučíková & Jan Votýpka

† These authors share first authorship.

*** Correspondence:** Oldřich Bartoš; e-mail: [124600@seznam.cz](mailto:124600@seznam.cz); Address: Military Health Institute, U Vojenske nemocnice 1200, Pavilion E1, Prague, 162 00, Czech Republic.

# Supplementary Methods

## PCR Analysis of Bartonella Adhesin A Deletion Phenotype

To confirm/refute the excision event, we designed specific primers that should bridge the putative deleted region. The forward primer sequence 5' CTGCATAATGTAGCTGCAGGATCG 3' and the reverse primer sequence 5' CATAGTAACGTAAGTTGGATACCGC 3' are expected to produce a 1972nt long PCR amplicon in case of the deletion, which we called BadA in our experiments. As an internal control of the PCR reaction and to check the presence of *Bartonella grahamii* subsp. *shimonis* subsp. nov. DNA in the host samples, we designed primers targeting the penicillin-binding protein gene with a unique *B. grahamii* subsp. *shimonis* subsp. nov. DNA sequence. The sequence of the forward primer 5' GTTTCAGGAGAAAGCGTAGAAGG 3' and the sequence of the reverse primer 5' CGCTGACATTAGGGTTGATAGG 3' lead to a 1855nt PCR amplicon, which we designated PBP. We used *Bartonella gliris* sp. nov. DNA in the analysis to prove that the primers specifically amplify only *B.* *grahamii* subsp. *shimonis* subsp. nov. DNA as we have expected.

All PCR reactions were performed on a PTC-200 DNA Engine Cycler (BioRad). In all PCR reaction programs, we used LongAmp® Taq 2X Master Mix (NEB) according to the manufacturer's instructions for thermocycling conditions. DNA input was 5 ng per reaction for bacterial colony samples, 50 ng for host samples. Bacterial colony DNA amplicons were analyzed after 22 cycles, host DNA amplicons after 45 cycles. The high number of cycles in the case of host DNA analysis were chosen based on the assumption of extremely low amounts of bacterial DNA in these samples. Here we reached the limits of the given reactions, and we were also limited by the very small volumes and DNA concentrations of the isolates of the host samples.

All PCR products together were analyzed by gel electrophoresis on a single 0.8% TBE agarose gel, stained with ethidium bromide, with 1kb DNA ladder (Promega) (see supplementary figure).

**1.2 Mass Spectrometry**

**Preparation of Whole Cell Lysates**

In total, 5 samples were analysed. Biological duplicates for *B. grahamii* subsp. *shimonis* subsp. nov. and *B. gliris* sp. nov. and one sample of *B. henselae* CNCTC 5656 were cultured for one week under the same conditions (37 °C, 5% CO2).

For each sample, several colonies were removed from the agar plate, resuspended in phosphate-buffered saline, and pelleted (7300g, 20 min, 4 °C), and then the washing step was repeated. The pellet was resuspended in 400 μl of Lysis Buffer (10% (w/v) sodium deoxycholate (SDC) in 50 mM NH4HCO3) and incubated at 95 °C for 10 min. The lysate was treated with benzonase (Sigma-Aldrich), 150 U/mL, for 10 min on ice. Unbroken cells were removed by centrifugation (12,000g, 10 min, 4 °C) and subsequent filter sterilization of the supernatant through a Millex-GP Syringe Filter Unit (0.22 μm, polyethersulfone, Millipore). The protein concentration was determined by a BCA assay (Sigma-Aldrich).

**Protein Extraction and Digest**

Protein extraction and digestion were provided according to a previously described protocol (Klimentova et al. 2021). Protein samples were adjusted to final concentration of 0.125 μg/μL using 100 mM NH4HCO3. Proteins were reduced with 10 mM dithiothreitol at 37 °C for 60 min and alkylated with 20 mM iodoacetamide at room temperature for 30 min in darkness, and the unreacted iodoacetamide was quenched with further 10 mM dithiothreitol at room temperature for 15 min. The samples were diluted with 50 mM NH4HCO3 to decrease the concentration of SDC to 0.5% and digested with sequencing grade trypsin (Promega) overnight at 37 °C. SDC was removed following the modified phase transfer protocol (Masuda et al. 2008). Briefly, ethyl acetate was added and the digested product was acidified by trifluoroacetic acid (TFA) to a final concentration of ca. 2% (v/v). The mixtures were vortexed vigorously, centrifuged at 10,000g for 1 min, and the upper organic layer was removed. The extraction was repeated twice with fresh ethyl acetate, and residual ethyl acetate was removed by 20 min of vacuum drying. The aqueous phases were then desalted on Empore C18-SD (4 mm/1 mL) extraction cartridges (Sigma-Aldrich), dried in vacuum, and stored at −40 °C until the analysis.

**LC-MS/MS Analysis**

An UltiMate 3000 RSLCnano system controlled by Chromeleon software (Dionex, USA) was used for chromatography separation. Each sample was loaded onto a PepMap100 C18, 3 µm, 100 Å, 0.075 × 20 mm trap column (Dionex) at 5 µL/min for 5 min. Peptides were separated on a PepMap RSLC C18, 2 µm, 100 Å, 0.075 × 150 mm analytical column (Dionex) by a gradient formed by mobile phase A (0.1% formic acid, FA) and mobile phase B (80% ACN, 0.1% FA), running from 4 to 34% in 68 min, and from 34 to 55% of mobile phase B in 21 min at a flow rate of 0.3 µL/min at 40 °C. Eluted peptides were on-line electrosprayed into Q-Exactive mass spectrometer using a Nanospray Flex ion source (Thermo Scientific). Positive ion full scan MS spectra (m/z 350-1650) were acquired using a 1×106 AGC target in the Orbitrap at 70 000 resolution. Top 12 precursors of charge state ≥2 and threshold intensity of 5×104 counts were selected for HCD fragmentation, with a dynamic exclusion window of 60 s. The isolation window of 1.6 Da and normalized CE of 27 was used. Each MS/MS spectrum was acquired at resolution of 17,500, with a 105 AGC target and a maximum 100 ms injection time.

**LFQ - Raw Data Processing of Bartonella Proteins**

Raw files acquired in LC-MS/MS analysis were further analyzed in MaxQuant (Cox et al. 2008) (version 2.1.2.0) and the Andromeda was used as search engine (Cox et al. 2011) to search the detected features against the *B. henselae*, *B. grahamii* subsp. *shimonis* subsp. nov. and *B. gliris* sp. nov. against custom proteomic databases. One database contained 1121 single copy orthologous sequences present in all species, nevertheless, all (sub)species were represented by their specific sequences. The second database consisted only of Bartonella Adhesin A(-like) proteins as they were identified in *B. grahamii* subsp. *shimonis* supsp. nov..

Only tryptic peptides that were at least seven amino acids in length with up to two missed cleavages were considered. The initial allowed mass tolerance was set to 4.5 ppm at the MS level and 0.5 Da at the MS/MS level. The oxidation of methionine was set as variable modification and carbamidomethylation of cysteine was set as a fixed modification. A false discovery rate (FDR) of 1 % was imposed for peptide-spectrum matches (PSMs) and protein identification using a target–decoy approach. Relative quantification was performed using the default parameters of the MaxLFQ algorithm (Cox et al. 2014) with the minimum ratio count set to 2.

**LFQ Data Analysis of Bartonella Proteins**

The “proteinGroups.txt” MaxQuant output file for each search were uploaded into Perseus (Cox et al. 2014) (version 2.0.3.1) for further filtering and statistical analysis. Decoy hits, proteins only identified by site, and potential contaminants were removed and log2 transformation of LFQ intensities was applied. Finally, the functional annotations available for Perseus on August 25th 2022 for Bartonella spp. were added.

**Supplementary References**

Cox, J., Hein, M. Y., Luber, C. A., Paron, I., Nagaraj, N., & Mann, M. (2014). Accurate Proteome-wide Label-free Quantification by Delayed Normalization and Maximal Peptide Ratio Extraction, Termed MaxLFQ. *Molecular & Cellular Proteomics*, *13*(9), 2513–2526. https://doi.org/10.1074/mcp.M113.031591

Cox, J., & Mann, M. (2008). MaxQuant enables high peptide identification rates, individualized p.p.b.-range mass accuracies and proteome-wide protein quantification. *Nature Biotechnology*, *26*(12), 1367–1372. https://doi.org/10.1038/nbt.1511

Cox, J., Neuhauser, N., Michalski, A., Scheltema, R. A., Olsen, J. V., & Mann, M. (2011). Andromeda: A Peptide Search Engine Integrated into the MaxQuant Environment. *Journal of Proteome Research*, *10*(4), 1794–1805. https://doi.org/10.1021/pr101065j

Klimentova, J., Rehulka, P., Pavkova, I., Kubelkova, K., Bavlovic, J., & Stulik, J. (2021). Cross-Species Proteomic Comparison of Outer Membrane Vesicles and Membranes of *Francisella tularensis* subsp. *Tularensis* versus subsp. *Holarctica*. *Journal of Proteome Research*, *20*(3), 1716–1732. https://doi.org/10.1021/acs.jproteome.0c00917

Masuda, T., Tomita, M., & Ishihama, Y. (2008). Phase Transfer Surfactant-Aided Trypsin Digestion for Membrane Proteome Analysis. *Journal of Proteome Research*, *7*(2), 731–740. https://doi.org/10.1021/pr700658q

# Supplementary Figures and Tables

## Supplementary Figures

**
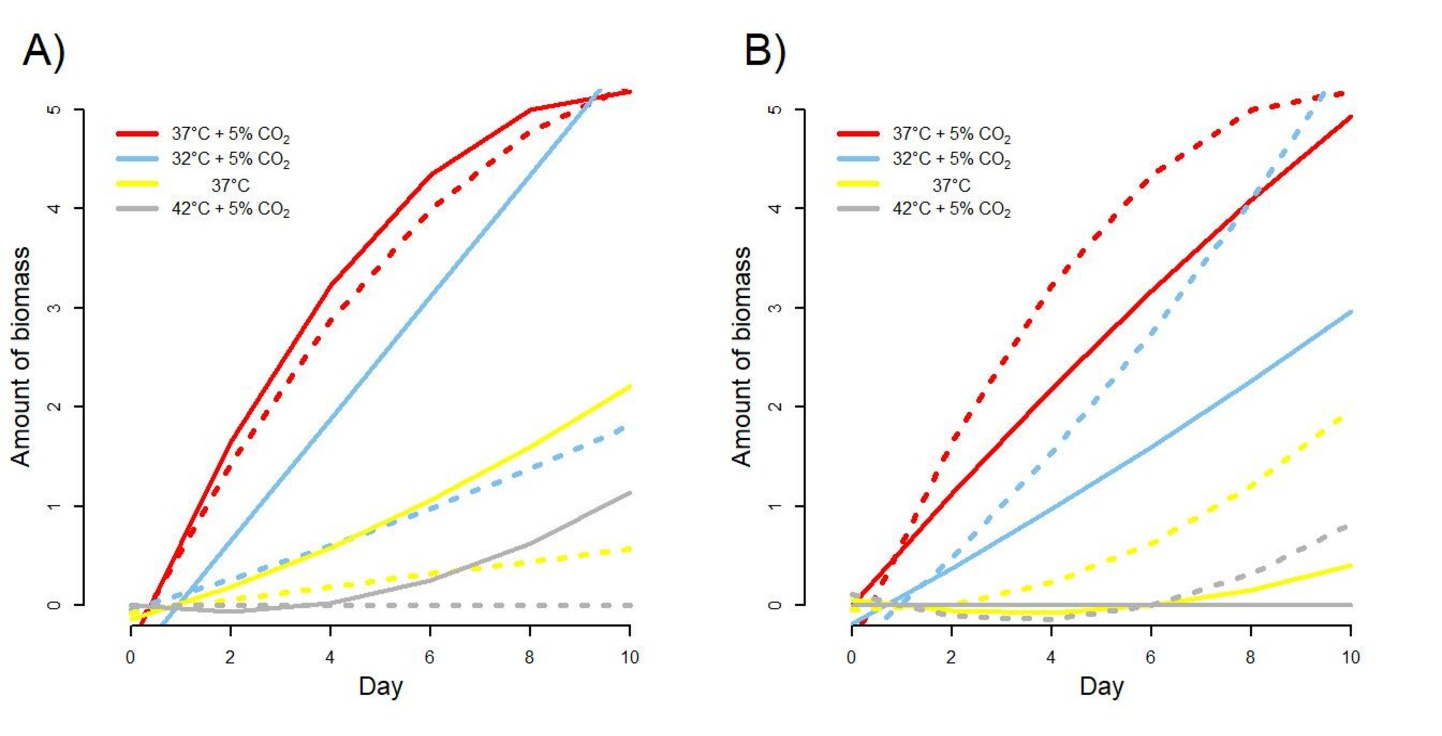
**

**Supplementary Figure 1:** The average biomass coverage for plates grown under different conditions (37, 32, 42 °C with 5% of CO_2_ or at 37 °C without added CO_2_) and evaluated over ten days of cultivation. **A**) *Bartonella grahamii* subsp. *shimonis* subsp. nov. represented by two strains (one is depicted with solid and the other with dashed lines). **B**) *Bartonella gliris* sp. nov. represented by two strains (solid and dashed lines). Plates were photographed every two days to evaluate the amount of biomass; a subjective scale from 1 (only a few small colonies visible) to 5 (fully grown colonies covering the plate) was used to assess the amount of biomass on the plates and to determine the growth rate under various conditions. Measured values were fitted to polynomial functions of second degree for the purposes of visualization.


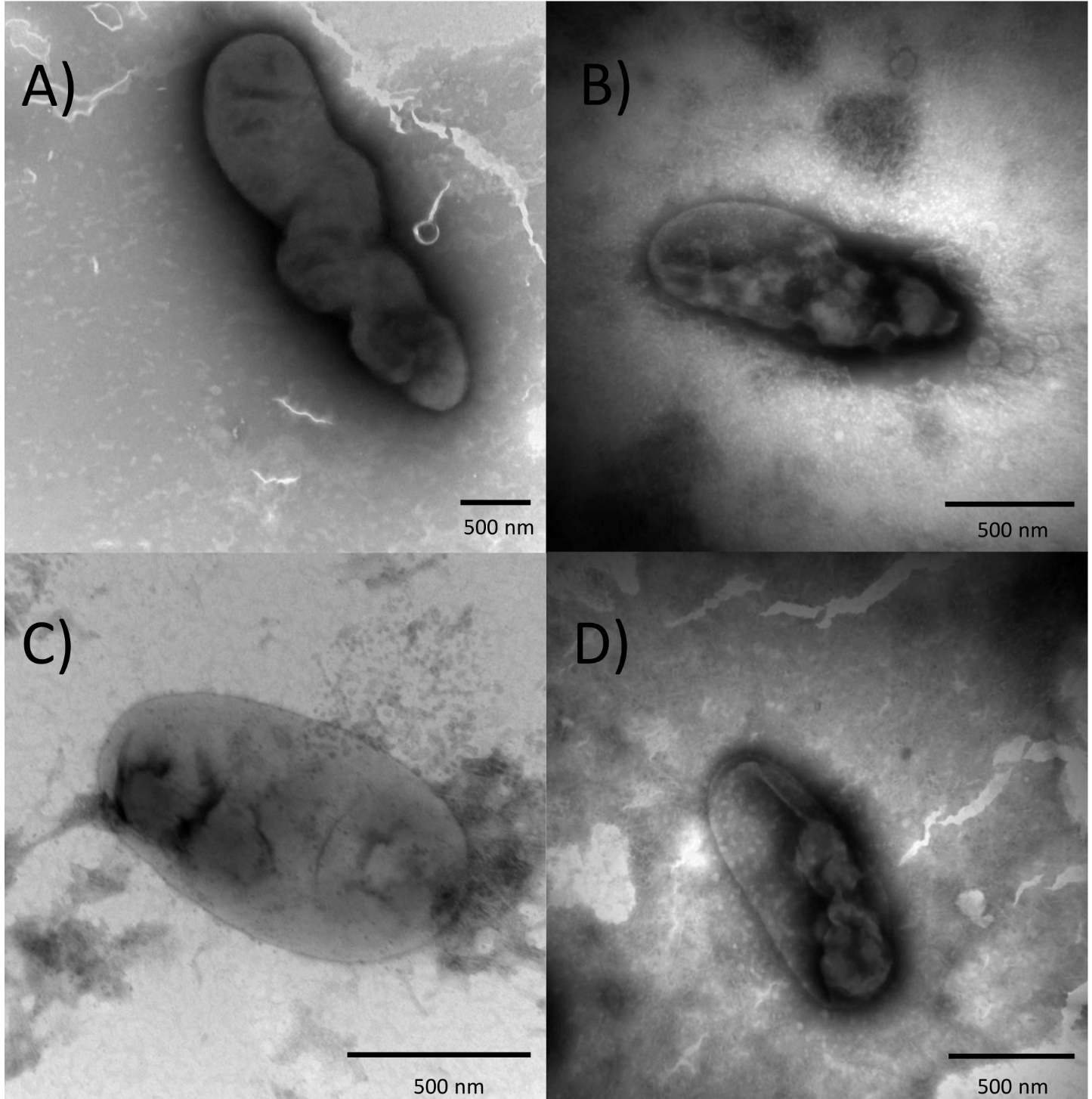


**Supplementary Figure 2**: The micrographs of: A) *B. gliris* sp. nov. strain GG20g1, B) *B. gliris* sp. nov. strain GG6g2, C) *B. grahamii* subsp. *shimonis* subsp. nov. strain GG3s1, and D) *B. grahamii* subsp. *shimonis* subsp. nov. strain GG23s2 prepared by negative staining by phosphotungstic acid and visualized by a transmission electron microscope (JEM 200CX Jeol).


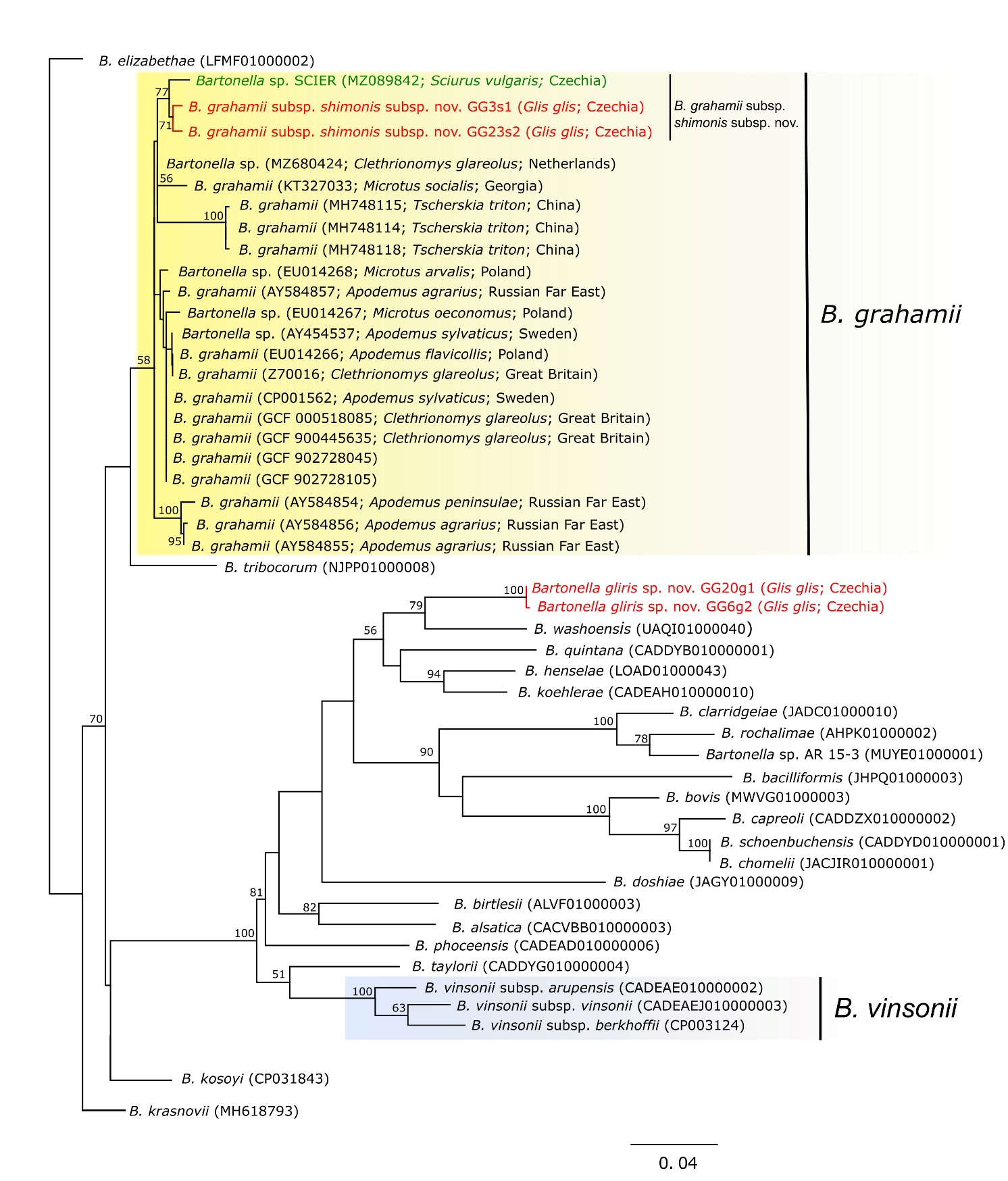


**Supplementary Figure 3:** The phylogenetic tree of the *Bartonella* species/strains based on the partial citrate synthase (*gltA*) gene (743 bp) sequences reconstructed using the Maximum likelihood method. Statistical support at the nodes is presented as bootstrap values (PhyML; bootstrap of 1000 replicates); the tree was rooted with the *B. elizabethae* sequence. Newly obtained strains are highlighted by red font color, a previously obtained sequence clustering into the novel subspecies *B. grahamii* subsp. *shimonis* subsp. nov. is highlighted by green font color. Background color indicates species-subspecies clusters.


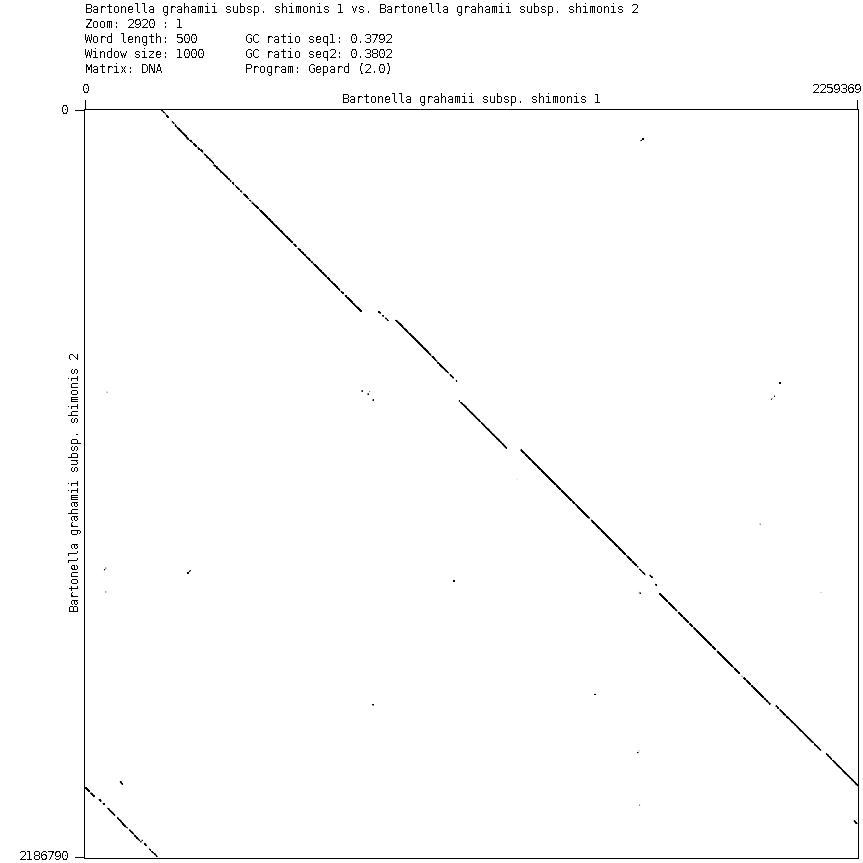


**Supplementary Figure 4:** Genome to genome dot-plot of two described *Bartonella grahamii* subsp. *shimonis* subsp. nov. strains. Significant breaks in collinearity correspond to described deletion events or to prophage sites.


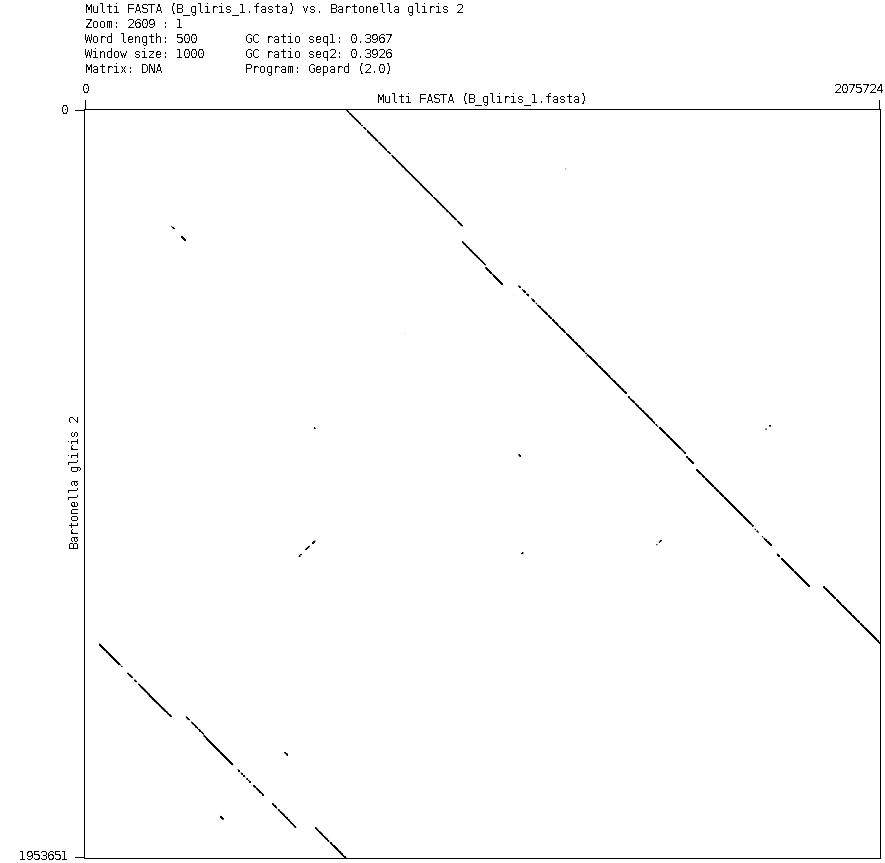


**Supplementary Figure 5:** Genome to genome dot-plot of two described *Bartonella gliris* sp. nov. strains. Significant breaks in collinearity correspond to prophage sites.

**
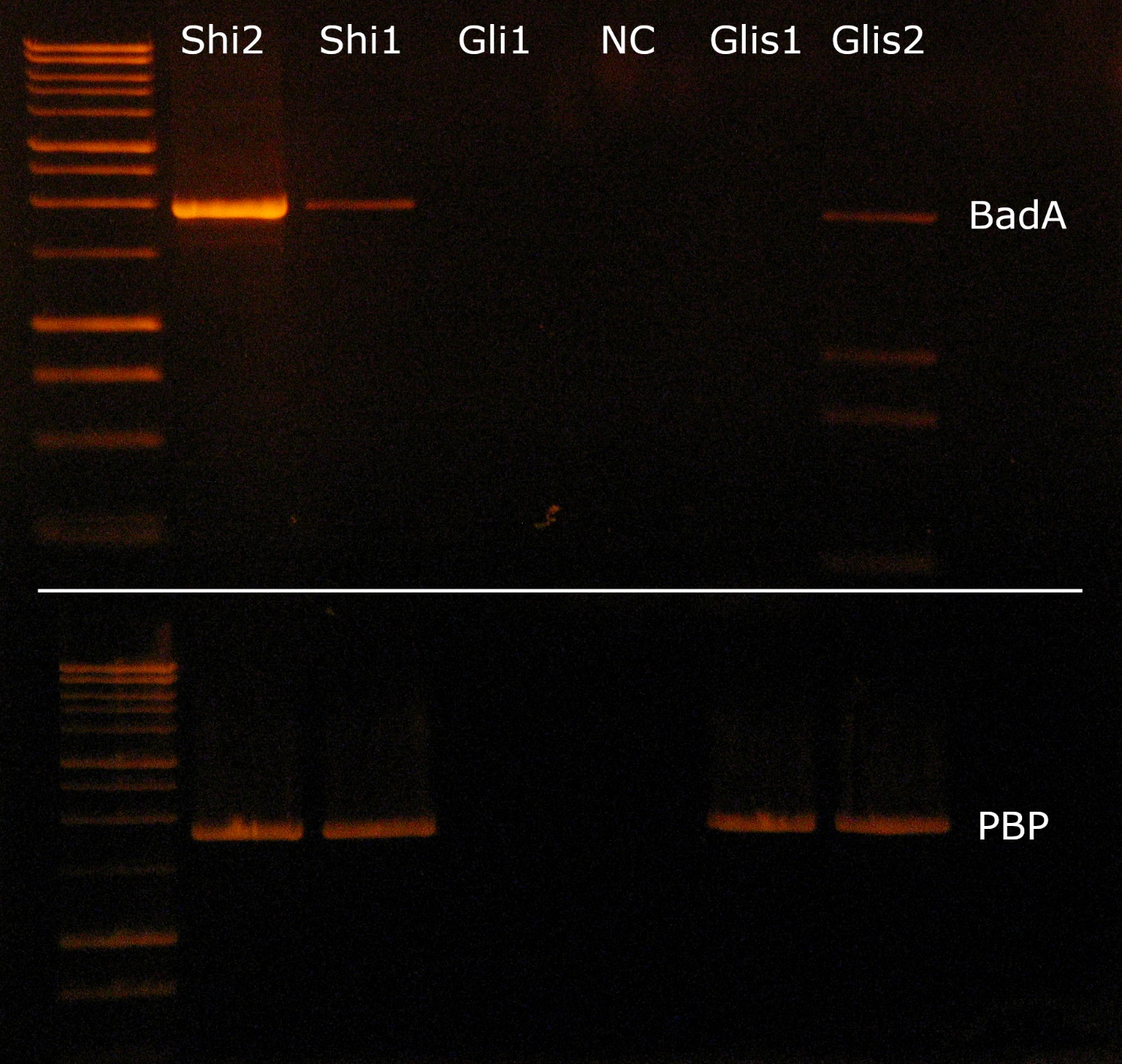
**

**Supplementary Figure 6**: Legend: Shi2 - *Bartonella grahamii* subsp. *shimonis* subsp. nov. strain GG23s2, Shi1 - *Bartonella grahamii* *shimonis* subsp. nov GG3s1, Gli1 - *Bartonella gliris* sp. nov. strain GG20g1, NC - negative control, Glis1 - host sample 1 (whole blood DNA of edible dormouse), Glis2 - host sample 2 (whole blood DNA of edible dormouse). The upper part of the figure represents the demonstration of the presence of an excision event in cultured *B. grahamii* subsp. *shimonis* GG3s1 and GG23s2, designated as BadA. The presence of weak bands in the Glis2 sample was considered as a false positive result, which was confirmed by sequencing of the products. The lower part of the figure shows the presence of penicillin-binding protein (PBP), specific for *B. grahamii* subsp. *shimonis*, used as an internal reaction control. Both markers in the most left column are 1kb DNA ladder (Promega).


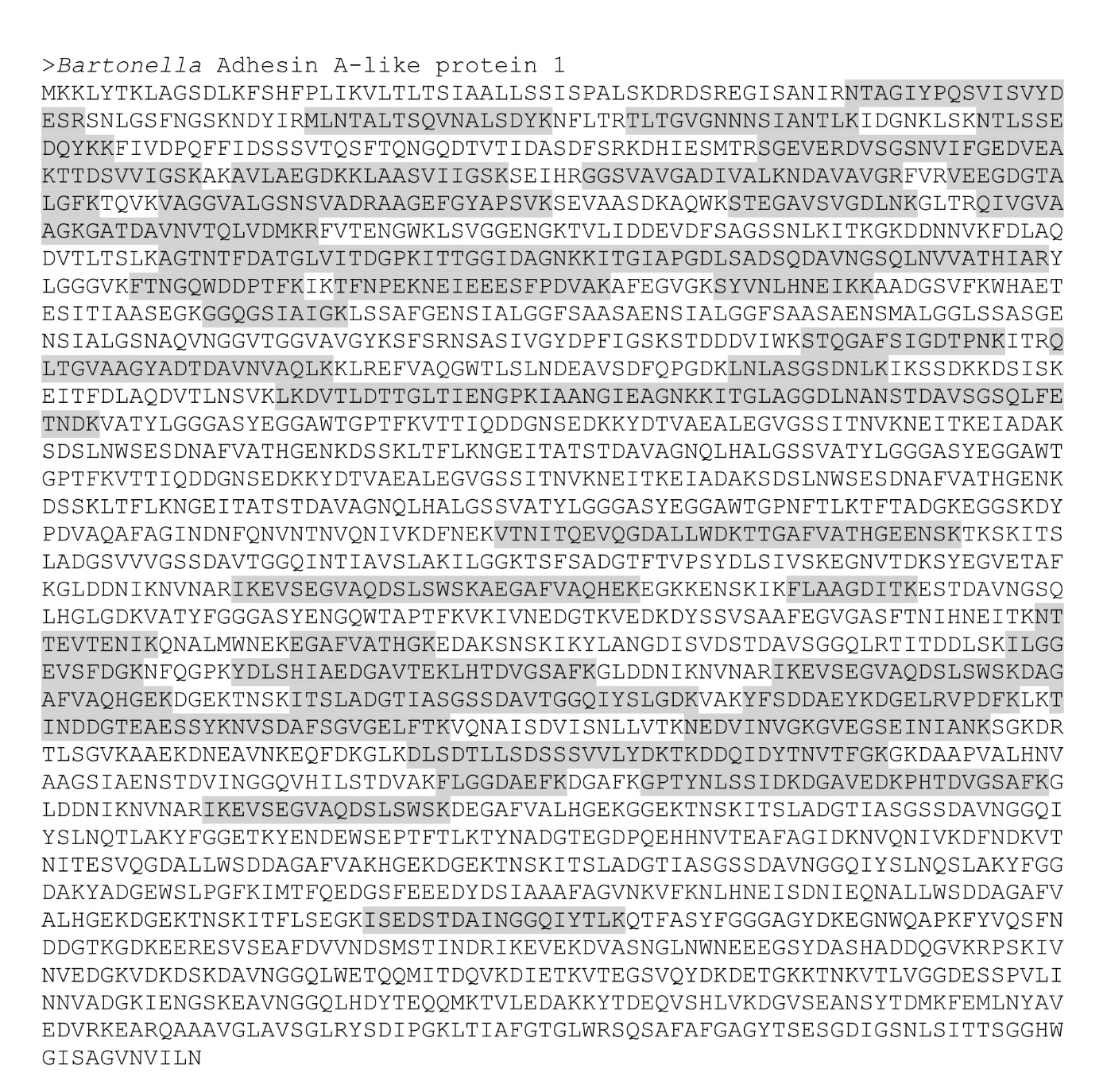


**Supplementary Figure 7**: Protein sequence of *Bartonella* Adhesin A-like protein 1: Sequences highlighted with gray color represent peptides detected by the proteomic analyses unambiguously originating from this particular protein.

## Supplementary Tables

| **Sample** | **ONT Total Reads** | **ONT Total Bases** | **ONT Reads Length N50** | **Illumina Reads Total** | **Illumina Reads Length** |
| --- | --- | --- | --- | --- | --- |
| **Bartonella grahamii subsp.** **shimonis** subsp. nov. GG3s1 | 14 535 | 59 710 146 | 5 944 | 1 496 863 | 150+150 |
| **Bartonella grahamii subsp. shimonis** subsp. nov. GG23s2 | 431 874 | 1 596 052 204 | 4 687 | 1 375 366 | 150+150 |
| **Bartonella gliris** sp. nov. GG20g1 | 68 052 | 283 879 933 | 5 513 | 1 064 962 | 150+150 |
| **Bartonella gliris**  sp. nov. GG6g2 | 53 273 | 257 460 561 | 6 679 | 1 527 680 | 150+150 |

**Supplementary Table 1**: Statistics of Oxford Nanopore Technologies (ONT) and Illumina sequencing results. Considering the ONT sequencing libraries, only reads ≥ 1000bp were considered.

| **Sample** | **NCBI accession number** |
| --- | --- |
| **Bartonella grahamii subsp.** **shimonis** subsp. nov. GG3s1 | NA |
| **Bartonella grahamii subsp. shimonis** subsp. nov. GG23s2 | NA |
| **Bartonella gliris** sp. nov. GG20g1 | NA |
| **Bartonella gliris**  sp. nov. GG6g2 | NA |
| **Bartonella apis** BBC0122 | GCF_002007565.1 |
| **Bartonella apis** PEBO149 | GCF_001952065.1 |
| **Bartonella australis** AUST/NH1 | GCF_000341355.1 |
| **Bartonella bacilliformis** ATCC 35685D-5 | GCF_001559035.2 |
| **Bartonella bovis** 91-4 | GCF_000384965.1 |
| **Bartonella clarridgeiae** 73 | GCF_000253015.1 |
| **Bartonella elizabethae** NCTC12898 | GCF_900638615.1 |
| **Bartonella grahamii** A1JPB | GCF_902728105.1 |
| **Bartonella henseale** BM1374163 | GCF_000612965.1 |
| **Bartonella melophagi** K-2C | GCF_000278255.1 |
| **Bartonella queenslandensis** BqUM | GCF_018155095.1 |
| **Bartonella quintana** MF1-1 | GCF_009936175.1 |
| **Bartonella rochalimae** ATCC BAA-1498 | GCF_000706645.1 |
| **Bartonella schoenbuchensis** R1 | GCF_002022685.1 |
| **Bartonella tamiae** Th307 | GCF_000279995.1 |
| **Bartonella tribocorum** BM1374166 | GCF_000689355.1 |
| **Bartonella vinsonii subsp. arupensis** ATCC 700727 | GCF_902825115.1 |
| **Bartonella vinsonii subsp. berkhoffi**i Winnie | GCF_000341385.1 |
| **Bartonella vinsonii subsp. vinsonii** CIP 103738 | GCF_902825235.1 |
| **Bartonella washoeensis** Sb944nv | GCF_000278135.1 |

**Supplementary Table 2**: List of samples included into the phylogenomic and PCA analyses; NCBI accessions are provided.

| **Species** | **Bartonella grahamii subsp.** **shimonis** subsp. nov. GG3s1 | **Bartonella grahamii subsp. shimonis** subsp. nov. GG23s2 | **Bartonella gliris** sp. nov. GG20g1 | **Bartonella gliris**  sp. nov. GG6g2 |
| --- | --- | --- | --- | --- |
| Bartonella_apis_BBC0122 | 0.691 | 0.690 | 0.693 | 0.692 |
| Bartonella_apis_PEBO149 | 0.690 | 0.690 | 0.692 | 0.690 |
| Bartonella_australis | 0.770 | 0.768 | 0.780 | 0.781 |
| Bartonella_bacilliformis | 0.783 | 0.783 | 0.794 | 0.794 |
| Bartonella_bovis | 0.790 | 0.791 | 0.799 | 0.802 |
| Bartonella_clarridgeiae | 0.785 | 0.786 | 0.796 | 0.798 |
| Bartonella_elizabethae | 0.876 | 0.879 | 0.824 | 0.825 |
| Bartonella_grahamii | 0.936 | 0.934 | 0.838 | 0.838 |
| Bartonella_henselae | 0.833 | 0.833 | 0.850 | 0.851 |
| Bartonella_melophagi | 0.790 | 0.790 | 0.803 | 0.803 |
| Bartonella_queenslandensis | 0.878 | 0.879 | 0.826 | 0.826 |
| Bartonella_quintana | 0.833 | 0.831 | 0.857 | 0.859 |
| Bartonella_rochalimae | 0.781 | 0.782 | 0.792 | 0.793 |
| Bartonella_schoenbuchensis | 0.787 | 0.788 | 0.800 | 0.800 |
| Bartonella_tamiae | 0.708 | 0.707 | 0.707 | 0.707 |
| Bartonella_tribocorum | 0.879 | 0.882 | 0.831 | 0.827 |
| Bartonella_vinsonii_arupensis | 0.835 | 0.835 | 0.843 | 0.842 |
| Bartonella_vinsonii_berkhoffii | 0.835 | 0.834 | 0.843 | 0.844 |
| Bartonella_vinsonii_vinsonii | 0.838 | 0.837 | 0.845 | 0.846 |
| Bartonella_washoensis | 0.831 | 0.833 | 0.886 | 0.889 |

**Supplementary Table 3**: Comparison of Average Nucleotide Identity estimates for all newly described strains against all other included *Bartonella* (sub)species.

| **Sample** | **Contigs** | **Total Bases** | **GC content [%]** |
| --- | --- | --- | --- |
| **Bartonella grahamii subsp.** **shimonis** subsp. nov. GG3s1 | 1 | 2 259 369 | 37.92 |
| **Bartonella grahamii subsp. shimonis** subsp. nov. GG23s2 | 1 | 2 186 790 | 38.02 |
| **Bartonella gliris** sp. nov. GG20g1 | 2 | 2 075 724 | 39.67 |
| **Bartonella gliris**  sp. nov. GG6g2 | 1 | 1 953 651 | 39.26 |

**Supplementary Table 4**: Basic assembly statistics including number of assembled contigs (none of them is considered to represent a plasmid), size of the genome and GC content.
